# Obesity effect on a multimodal physiotherapy program for low back pain suffers: patient reported outcome

**DOI:** 10.1186/1745-6673-8-13

**Published:** 2013-05-10

**Authors:** Antonio I Cuesta-Vargas, Manuel González-Sánchez

**Affiliations:** 1 Department of Physiotherapy, Faculty of Health Sciences, University of Malaga, Malaga, Spain; 2 School of Clinical Sciences, Faculty of Health at the Queensland University of Technology, Queensland, Australia

**Keywords:** Obesity, Low back pain, Physiotherapy, Disability, Health-related quality of life

## Abstract

**Background:**

Several studies have linked obesity to the increased likelihood of lower back pain, but there are no studies focussing on the effectiveness of a multimodal physiotherapy programme (MPP) in obese subjects who suffer from chronic non-specific lower back pain (CNLBP). The aim of this study was to compare the effectiveness of an MPP in obese (G_1_) (body mass index (BMI):≥30) and non-obese (G_2_) (BMI:<30) patients with CNLBP.

**Methods:**

A quasi-experimental study with pre- and post-intervention evaluations of an MPP (lasting 8 weeks) was conducted on obese and non-obese patients with CNLBP. A total of 53 people were included in the study: G_1_, composed of 19 patients (10 men and 9 women) with a BMI of 33.75 and a mean age of 52.94 years, and G_2_, composed of 34 patients (18 men and 16 women) with a mean age of 49.19 years and an average BMI of 25.56. All patients were measured to calculate pre-intervention (baseline) and post-intervention (8 weeks) changes in disability (RMQ) and health related quality of life in physical and mental health component state of SF12 and quality of life (EQ-5D and EQ-VAS).

**Results:**

Post-intervention, non-obese group shown significant high improve than obese group in disability (RMQ: 4.00), physical component state of SF-12: (-7.26) and quality of life (EQ-VAS.: -10.49).

**Conclusions:**

In patients with CNLBP, a BMI more than or equal to 30 minimises the effects of an MPP lasting 8 weeks.

## Introduction

Low back pain (LBP) and obesity are two of the most important public health concerns [1-3]. LBP is considered to be a biopsychosocial phenomenon, its prevalence throughout a lifespan being between 60 and 85% [2]. The number of overweight or obese people has shown a global increase over recent years, especially in industrialised countries [3].

The hypothesis of Mortimer et al. [4] that there is a relationship between obesity and an increased chance of having LBP is supported by recent studies [4,5]. Since this increase is directly proportional to the body mass index (BMI) [5], it has been proposed that obesity could be considered an instrument for predicting LBP [4].

A subsequent study confirmed this relationship and found that the degree of correlation persisted after adjustments were made based on other factors such as smoking, education, type of work and time spent on physical activity [5].

It has been noted that obesity is associated with changes in the vertebral plates [6], degenerative changes in the intervertebral disc and decreased spinal mobility [6,7]. People with high BMI scores have lower levels of functionality, which is further exacerbated by LBP [8].

In the treatment of LBP, multimodal interventions [9] (the combination of different techniques within the same treatment programme) have been proven to be more effective than a single technique. Within passive treatments, manual therapy (MT) is a more effective intervention in reducing short-term pain [9]. On the other hand, active treatments such as therapeutic exercise (TE) show strong evidence compared to standard medical practice (such as anti-inflammatory drugs and painkillers) of reducing pain and disability in non-specific LBP (NLBP) [9]. It has been found that health education (HE) interventions prior to and during treatment allow a re-conceptualisation of the problem by the patients themselves, which increases their level of commitment and involvement in the recovery process [10].

The combination of MT, TE and HE interventions on lower back pain has been shown to be effective in subjects with normal BMI [11,12], displaying improvements in pain, functional ability and general health [11,12].

Several studies have linked obesity to the increased likelihood of LBP, but no studies have studied the effectiveness of a multimodal physiotherapy intervention in obese subjects with chronic NLBP (CNLBP) [13].

The aim of this study was to analyse the effectiveness of a multimodal physiotherapy programme (MPP) lasting 8 weeks on obese and non-obese patients with CNLBP.

The initial hypothesis was that obese patients would respond less to the MPP than non-obese patients.

## Material and methods

Design: A quasi-experimental study with pre- and post-intervention evaluations of an MPP lasting 8 weeks conducted on two groups: obese and non-obese patients suffering from CNLBP.

Subjects: The patients in this study had CNLBP and were divided into two groups according to their BMI scores: obese (G_1_) and non-obese (G_2_). The patients with BMI scores equal to or greater than 30 were placed in the obese group according to the rates recommended by the World Health Organization (WHO), using the following formula: BMI = weight (kg)/height^2^ (m) [14].

Inclusion criteria for the patients were CNLBP without radiation to the lower limbs and lasting for more than twelve weeks. The exclusion criteria were: patients who refused to participate, those suffering pain in the spine following a specific lumbar spinal pathology, those with nerve root/radicular pain, individuals with pain processes, patients who had an infection, neoplasm, metastasis, osteoporosis, arthritis, or fractures, those who showed cognitive impairment of any etiology and exercise intolerance with any cause and to has introduced the fact you have undergone surgery in the past 12 months [8]. Patients whose pre-intervention rate in the Roland Morris Questionnaire (RMQ) [15] was less than 7 and greater than 13 did not participate in the study. The intention was to recruit patients with a medium level of disability, following the statement made by Woby et al. (2004) [16] that the level of disability was between the two limits (7 and 13) established in the exclusion criteria. In addition, we excluded all the obese participant that during the treatment period developed a hypo caloric diet.

**Figure 1:**
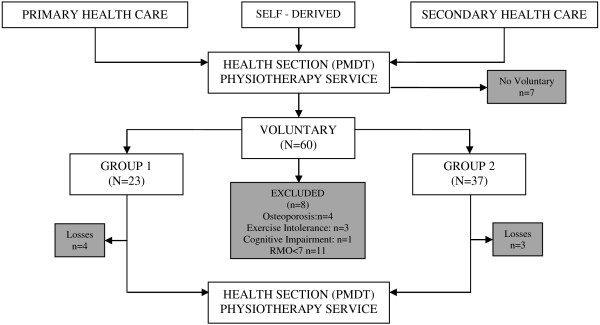
Description patient diagram.

In this study, the number of participating subjects was 60. A total of seven patients were excluded during the intervention (four in G_1_ and three in G_2_). Three subjects were excluded in the first group due to a lack of adherence to treatment and one was lost due to changes in their work schedule. In the second group, two participants left because of changes in their work schedules that made their participation impossible and one was excluded due to a lack of adherence to treatment. Thus, the total number of individuals who completed the intervention was 53 (Figure 1). The attendance of the patients was monitored via a roll call at the beginning of each session. The intervention groups were created based on BMI scores (G_1_ BMI < 30 kg/m^2^ and G_1_ BMI ≥ 30 kg/m^2^), as well as the availability of the patients. Thus, both obese and non-obese patients took part in the same intervention group.

Data from patients who did not attend at least 80% of the sessions were discarded due to the lack of adherence to treatment. The results are presented according to the intention to treat analysis.

Variables: The outcome variables were divided into primary scales (disability, measured by Roland Morris Questionnaire (RMQ)) and secondary scales: general health state, measure through the questionnaire Short Form 12 (SF-12), divisible in physical component summary scale (PCS), mental component summary scale (MCS), and quality of life (QoL) measure through the questionnaire EuroQol-5D (EQ5D and EQ-VAS).

The patients completed self-administered questionnaires before (baseline) and after the intervention (8 weeks). The questionnaires used were: two generic tools health related quality of life, the SF-12 and EuroQoL-5D and one specific tool for disability of low back pain, RMQ. The SF-12 [17], adapted from the long version SF-36 [18] and validated by Luo et al. (2003) with a test-retest reliability of 0.70; the EuroQol-5D [19] was validated in Spanish by Badia et al. (1999) [20], with a test-retest reliability 0.88 and the RMQ validated by Roland and Morris (1983) using the Spanish version validated by Kovacs et al. (2002) [21] with a test-retest reliability of 0.87.

Intervention programme: The MPP consisted of the following: individual assessments were performed using the ASETER 2.0 [22] software. During the interview, the active participation of the patient, and the importance of compliance and adherence to the programme were encouraged. Additionally, a LBP Decalogue (10 basic suggestions for back health education) was given, which was included as brief advice given by the experts during the explanation of the exercises. For 8 weeks, the patients took part in an MPP that aimed to improve basic physical abilities. This consisted of 60-minute sessions (an individualised programme was designed involving five-ten minutes of mobility exercise, five ten minutes training of motor control, 20 minutes muscle resistance and 20 minutes of strength exercises), in which the following objectives were targeted: improvement in the motor control of the local system and improvement in endurance and strength of the trunk musculature as well as in joint mobility. The intervention was supervised by three therapists with a minimum of 8 years of experience in the field of musculoskeletal disease with specific training in MT, TE and HE. The intervention groups were created based on BMI scores, but were also affected by the availability of the participants, so that the group undergoing an intervention could have contained obese and non-obese patients.

The improvement in joint mobility was performed by MT and stretching. First, the physiotherapist overcame the hypo-mobile barrier manually, and then the patient performed self-stretching exercises. Always starting from the same side, the patient performed a continuous stretch of 30 seconds, repeated it three times on each side, with a 30-second rest period between the sets. The sequence of muscle stretches was always the same, starting with the hip extensors, followed by the knee flexor muscles and lateral hip rotators and ending with ilio-lumbar stretching. When the participant achieved normal mobility, they then moved on to obtain the other improvements.

A load control system, which guided the movement, was used for leading functional movements to improve the muscle strength of the trunk. The intervention were focused on the musculature involved in everyday actions, such as climbing or descending stairs, standing up, and pulling and pushing. The load at the start was 50% of 1 Max.

Repetition: The sequence comprised of two sets of 15 to 20 repetitions with a 2-minute rest period between each. While performing the exercises, the patient was taught to stay focused on the local system in the neutral position by maintaining the lumbopelvic region [23].

The methodology for enhancing isometric muscle strength was based on proprioception exercises performed on a Swiss ball with an approximate diameter of the distance between the shoulder and the wrist of each participant. Each exercise was performed three times for 30 seconds with a 30-second rest period between the sets.

The principles of confidentiality and autonomy were maintained for each subject. Prior to the intervention, written informed consent was required and anonymity was guaranteed at all times. The Committee on Ethics and Research at the Faculty of Health Science, University of Malaga, approved this study.

Statistical analysis: Data were analyzed using the SPSS package (version 17.0). Mean and standard deviations or 95% confidence intervals of the values were calculated for each variable. The Kolmogorov-Smirnov test showed a normal distribution of the data (P > 0.05). Pre-intervention values prior to each condition were compared using the independent t-tests for continuous data. A 2x2 mixed model ANCOVA with BMI as the between-subjects variable and time, Separate ANCOVAs were performed with each dependent variable, using BMI as factor. The Bonferroni test was used for post hoc analysis. A P-value < 0.05 was considered statistically significant.

## Results

Two intervention groups participated in this study. G_1_ with 19 patients (10 men and 9 women) presented a mean age of 52.94 years (±10.53) and an average BMI score of 33.75 (±2.47). G_2_ formed by 34 patients (18 men and 16 women) with a mean age of 49.19 years (±14.88) and a mean BMI score of 25.56 (±3.09). Into the age of the participants, no significant differences were observed between groups.

After analysis of the standard deviation of each variable, only the variable EQ-5D presented a nonparametric distribution.

Table 1 shows an ANCOVA post intervention using obesity as factor. All variables showed significantly negative influence of obesity. The variable least affected was the SF12-PCS (Partial Eta Squared = 0.098), while the most influenced was RMQ (Partial Eta Squared = 0.546).

**Table 1:** ANCOVA analysis between groups

**Source**	**Type III Sum of Squares**	**F**	**Sig.**	**Partial Eta squared**	**R Squared**
**SF12-PCS (0-100)**	497.949	6.197	0.016	0.098	0.225
**SF12-MCS (0-100)**	1573.104	14.028	0.000	0.198	0.200
**EQ-5D (0-1)**	0.641	21.757	0.000	0.263	0.268
**EQ-VAS (0-100)**	4613.223	13.325	0.001	0.192	0.202
**RMQ (24-0)**	897.690	71.075	0.000	0.546	0.597

Obesity were used as factor.

**RMQ**: Roland Morris Questionnaire.

**SF12-PCS**: Physical Component State.

**SF12-MCS**: Mental Component State.

**EQ-5D**: Quality of Life Index.

**EQ-VAS**: Visual Analogic Scale Quality of life.

Furthermore, a paired sample T-Student test for the obese and non-obese groups was developed. In the obese group, highly significant improvements in disability (p ≤ 0.001), physical component summary scale (p ≤ 0.001) and quality of life (EQ-VAS) (p ≤ 0.001) were observed, with improvement rates of 5.00 (on a scale of 0 to 24), 8.68 (on a scale of 0 to 100) and 17.67 (on a scale of 0 to 100), respectively (Table 2).

**Table 2:** Pretest-posttest differences in obese and non-obese participants (t-tests for paired samples)

	**G**_**1**_**: BMI ≥30**			**G**_**2**_**: BMI <30**		
	**(95% CI)**			**(95% CI)**		
	**Pre TEST**	**Post TEST**	**Mean Difference**	**Pre TEST**	**Post TEST**	**Mean Difference**
**RMQ**	**12.60**	**7.60**	5.00***	**12.34**	**3.6**	8.74***
(24-0)	(8.97/15.64)	(9.17/6.03)	(5.80/4.20)	(14.72/9.80)	(9.27/0.43)	(11.67/2.86)
**SF12-PCS**	**41.74**	**50.42**	−8.68***	**41.3**	**57.68**	**−16.38**
(0-100)	(32.12/51.27)	(63.51/39.90)	(-13.14/-4.21)	(48.12/35.10)	(64.38/45.71)	(-24.14/2.03)
**SF12-MCS**	**46.97**	**50.97**	**−4.00**	**52.94**	**53.56**	**−0.62**
(0-100)	(55.01/38.86)	(61.55/38.21)	(-9.39/1.40)	(64.28/35.02)	(58.10/51.80)	(-1.42/0.07)
**EQ-5D**	**0.60**	**0.68**	**−0.08**	**0.72**	**0.7**	0.02**
(0-1)	(0.71/0.46)	(0.78/0.59)	(-0.17/0.02)	(0.66/0.91)	(0.78/0.61)	(-0.40/0.06)
**EQ-VAS**	**61.33**	**79.00**	−17.67***	**64.74**	**89.49**	−24.75**
(0-100)	(72.67/52.67)	(87.23/66.83)	(-23.72/-11.61)	(54.20/75.80)	(94.39/75.06)	(-28.16/-19.94)

Significance.

* ≤ 0.05.

** ≤ 0.005.

*** ≤ 0.001**.**

**RMQ**: Roland Morris Questionnaire (high RMQ values represent high disability).

**SF12-PCS**: Physical Component State.

**SF12-MCS**: Mental Component State.

**EQ-5D**: Quality of Life Index.

**EQ-VAS**: Visual Analogic Scale Quality of life.

The improvement in disability experienced by the obese group was highly significant (p ≤ 0.001), while that in the quality of life was moderately significant (EQ-5D p = 0.003; EQ-VAS p = 0.002), with improvement rates of 8.74 (on a scale of 0 to 24), 0.02 (on a scale of 0 to 1) and 24.75 (on a scale of 0 to 100), respectively (Table 2). In addition, no significant differences were observed in the BMI between pre and post-intervention into the groups.

## Discussion

Given the results, the assumed hypothesis that the effectiveness of an MPP was significantly lower in people with CNLBP and a BMI score above 30 (obese) could be accept. This confirmation was provided by the differences in the primary outcome variable (RMQ) and the general state of physical health between the obese and non-obese groups.

The objective of this study was not to directly seek a change in the BMI scores, but rather to observe the effectiveness of treatment in this population. Only one published study was found in the literature analysing the effect of an MPP in patients suffering LBP with a BMI score above 30 [13].

The cut-off point between obesity and non-obesity, established by the WHO as a BMI score of 30 for obesity. It has been proven that there is a greater tendency for obese people to suffer from LBP [24]. Furthermore, it is at this cut-off point that the relationship between the BMI score and the potential risk of LBP becomes stronger [24].

Using ANCOVA, where obesity was used as a factor, it’s possible to observe the primary outcome variable (disability, assessed by the RMQ) is the variable that more undergoes the negative effect of obesity after 8 weeks of intervention (Table 1). However, all variables used suffer negative effects as a consequence of obesity. This reduced impact on the health related quality of life and quality of life may be due to these variables are more general than RMQ to measure change in patients suffering from low back pain. The fact that everyone has had a negative effect due to obesity, should lead to reflect on the possible relationship between the primary outcome variable (RMQ) and the other variables analyzed in this study (SF12-PCS, SF12-MCS, EQ-5D and EQ-VAS).

These results are in contrast to the conclusion reached by Mangwani et al. (2010) [13], who stated that BMI did not influence the effectiveness of a physical therapy programme specifically targeted at subjects suffering from CNLBP.

In the study by Mangwani et al. (2010) [13], the authors did not present the size of the effect or the mean differences of the independent sample analysis, but only the intra-group differences. Thus, the magnitude or direction of the effect was not revealed [25]. However, the results of the present study are clinically relevant for the group of non-obese subjects. It is for this reason that a subgroup of obese people with CNLBP might benefit from a specific strategy that is different to that aimed at non-obese CNLBP patients. In the paired sample analysis, significant improvements in the primary outcome variable (disability, as assessed by the RMQ) as well as in the SF12-PCS and EQ-VAS variables were found. In the disability variable, there was an improvement of 20.83% (5.00 out of 24) compared to the 4.46% observed in a study investigating the effects of a specific physiotherapy programme for LBP according to BMI scores [26]. That same study showed a 22.07% reduction in pain. This variable tends to have a large impact on QoL [25]; hence, it could be compared to the improvement of 17.67% observed in the EQ-VAS of the current study patients after 8 weeks of intervention.

Although the relationship between low back pain and obesity have been demonstrated in different studies [4,5], studies examining the effects of a physical therapy program in this population are rare. The effects of an MPP have been studied more frequently in populations where the BMI does not reach levels of obesity. These results are comparable to those of previous studies in which intra-group differences were observed [11,12]. In the primary outcome variable (disability measured by RMQ), the values obtained after 8 weeks of intervention with an MPP were highly significant; the decreasing disability levels observed here were similar to those obtained from previous trials, i.e., -8.74 (95% CI: -11.67 to -2.86) noted by us was comparable to 5.2 (95% CI 3.6 to 6.7) [11], 3.9 (95% CI 2.0 to 5.8) [9] and 3.0 (SD: ±4.8) [10].

Moreover, the post-intervention SF12-PCS value was highly significant and the size of the effect was comparable to those of previous studies, i.e., -16.38 (95% CI -24.14 to -0.07) is in line with the 8.93 (± 13.04) [11] and 10.6 (SD: ±12.9) [11] obtained in previous trials.

On other hand, if the impact that pain has on QoL was consider, the reductions in the quality of life may be related to a more general index. The QoL of the obese group underwent significant improvements of 0.02 (EQ-5D) and 24.75 (95% CI -28.16/-19.94) (EQ-VAS), which can be compared to the reductions in pain of 16.46 (±24.44) (VAS) [12].

Two intervention groups in order to investigate the cause of the differences in the results were compared. In the inclusion criteria, many similarities between the two trials (current study and Cuesta-Vargas 2010 [12]) were found; however, it was impossible to compare the interventions since the data provided by the aforementioned study did not allow this. After the end of the current study and the subsequent analysis of the results, in the group of subjects with a BMI score greater than 30, and therefore classified as being obese, response to the 8-week MPP intervention was less effective. Thus, it is important to study new strategies to increase the responsiveness of these subjects to interventions that focus on improving CNLBP. These results should support specific strategies that can increase the effect size in this subgroup. Due to the fact that obesity increases the risk of LBP, MPP surprising that more has not been studied in obese poputlations.

The main weakness of this study was using a single cut-off point in the BMI to determine the distribution of the groups. Future studies could verify how people at different levels of this anthropometric index (underweight, normal weight, overweight and obese) respond to intervention protocols based on treating CNLBP. In addition, given the prevalence of CNLBP and obesity, the sample size of the present study appears to be relatively small. Also, no a-priori statistical power calculations have been reported. Conversely, the main strength of this study was comparing pre- and post–intervention results demonstrating the differences in response to treatment in obese and non-obese CNLBP patients.

## Conclusions

The effects of an 8-week MPP, which combined MT, TE and HE, to treat patients suffering from CNLBP were reduced in those with a BMI greater than 30 and therefore classified as obese according to the WHO.

## Abbreviations

BMI: Body mass index; CNLBP: Chronic non-specific lower back pain; EQ-5D: EuroQoL 5 Dimensions; EQ-VAS: EuroQoL Visual Analogue Scale; HE: Health education; LBP: Low back pain; MCID: Minimum Clinically Important Diference; SF12-MCS: Mental component summary scale; MPP: Multimodal physiotherapy program; MT: Manual therapy; NLBP: Non specific low back pain; SF12-PCS: Physical component summary scale; QoL: Quality of life; RMQ: Roland Morris Questionnaire; TE: Therapeutic exercise; WHO: World Health Organization.

## Competing interests

The authors declare that they haven’t any commercial relationships which may lead to a conflict of interests.

## Authors’ contributions

AIC-V: have been involved designing and developed the conception of the studio, acquiring data, analyzing and developing the interpretation of data. This author has been involved in drafting the manuscript and he has given final approval of the version to be published too. MG-S: have been involved analyzing and developing the interpretation of data and drafting the manuscript. Both authors read and approved the final manuscript.
